# Readmission of Patients to Acute Psychiatric Hospitals: Determining Factors and Interventions to Reduce Inpatient Psychiatric Readmission Rates.

**DOI:** 10.1192/j.eurpsy.2023.1905

**Published:** 2023-07-19

**Authors:** E. Owusu, N. Nkire, F. Oluwasina, M. A. Lawal, V. I. Agyapong

**Affiliations:** 1 Department of Psychiatry, University of Alberta, Edmonton; 2 Department of Psychiatry, Dalhousie University, Halifax, NS, Canada

## Abstract

**Introduction:**

Appropriate and adequate treatment of psychiatric conditions in the community or at first presentation to the hospital may prevent rehospitalization. Information about hospital readmission factors may help to reduce readmission rates.

**Objectives:**

The scoping review sought to examine the readmission of patients to acute psychiatric hospitals to determine predictors and interventions to reduce psychiatric readmission rates.

**Methods:**

A scoping review was conducted in eleven bibliographic databases to identify the relevant peer‐reviewed studies. Two reviewers independently assessed full‐text articles, and a screening process was undertaken to identify studies for inclusion in the review. PRISMA checklist was adopted, and with the Covidence software, 75 articles were eligible for review. Data extraction was conducted, collated, summarized, and findings were reported.

**Results:**

The outcome of the review shows that learning disabilities, developmental delays, and alcohol, drug, and substance abuse, were crucial factors that increased the risk of readmission. It was also established through the review that greater access to mental health services in residential treatment and improved crisis intervention in congregate care settings were indicated as factors that reduce the risk of readmission.

**Conclusions:**

High rates of readmission may adversely impact healthcare spending. This study suggests a need for focused health policies to address readmission factors and improve community‐based care.

**Disclosure of Interest:**

None Declared

